# *Propionibacterium acnes* in Human Health and Disease

**DOI:** 10.1155/2013/493564

**Published:** 2013-12-24

**Authors:** Andrew McDowell, Sheila Patrick, Yoshinobu Eishi, Peter Lambert, Anne Eady

**Affiliations:** ^1^Centre for Stratified Medicine, School of Biomedical Sciences, University of Ulster, Altnagelvin Hospital, Londonderry BT47 6SB, UK; ^2^Centre for Infection and Immunity, School of Medicine, Dentistry and Biomedical Sciences, Queen's University, Belfast BT7 9BL, UK; ^3^Department of Human Pathology, Tokyo Medical and Dental University, Yushima, Bunkyo-ku, Tokyo 113-8519, Japan; ^4^School of Life and Health Sciences, Aston University, Aston Triangle, Birmingham B4 7ET, UK; ^5^Department of Dermatology, Harrogate and District NHS Foundation, Harrogate HG2 7SX, UK

While the association of the Gram-positive anaerobic bacterium *Propionibacterium acnes* with the inflammatory skin condition acne vulgaris has been known for over a century, its potential role in other human infections and clinical conditions has undoubtedly been underestimated. This is likely to reflect inadequate anaerobic processing methods and culture of clinical specimens (>7d incubation), combined with the traditionally held view that *P. acnes* in a biological sample simply reflects contamination from the resident skin microflora or is clinically irrelevant due to a low level of virulence [1]. On such basis, it is likely that an unknown number of *P. acnes* infections go undiagnosed and unrecognised. In addition, subtle clinical signs and symptoms that are often associated with *P. acnes *infections may further lead to the conclusion that an infection is absent, particularly in relation to prosthetic joints and other indwelling medical devices [2]. Despite the historical failure to recognise the pathogenic potential of *P. acnes*, there is now a growing awareness, as evidenced in the scientific literature, that the bacterium is an important cause of opportunistic human infection and should not be readily dismissed as a contaminant/passive presence in clinical samples [2–6]. Within the last decade, advances in our understanding of the population genetic structure of *P. acnes* combined with whole genome sequence (WGS) data now available for a large number of strains, mostly derived from the Human Microbiome Project, have provided a platform for hypothesis-driven research into the exact role of *P. acnes* in human health and disease and the possibility of developing vaccines and other novel therapeutic treatments [7–11]. In particular, the availability of WGS information has enabled both genomic and transcriptomic differences between lineages from phylogroups associated mainly with health or disease to be examined, thus identifying potential genetic determinants and mechanisms of virulence [11, 12]. Despite its ability to behave as an opportunistic pathogen, it must not be forgotten that *P. acnes* is part of the normal human microbiota and, consequently, also plays a role in human health via occupation of niches that could be colonized by other, more pathogenic, microorganisms. Furthermore, the powerful immunomodulatory properties of *P. acnes* have long been investigated for their capacity to stimulate protective host responses against various human cancers, and more generally against Th-2-mediated diseases. In light of the growing interest and recognition of the role of* P. acnes *in human health and disease, this special issue aims to provide a description of recent developments and advances in different areas of *P. acnes* research via original research and review articles, and thus provide a framework to discuss and identify key issues that need to be addressed in future studies.

Developments in our understanding of *P. acnes* bacteriophages are reviewed in this special issue by H. Brüggemann and R. Lood. They describe both historical and recent developments in the field and highlight how genomic analyses have provided insights into *P. acnes* phage biology, as well as the identification of phage sequence signatures in clustered regularly interspaced short palindromic repeats (CRISPRs) in *P. acnes*. They also discuss bacteriophages as a therapeutic strategy to combat *P. acnes*-associated diseases, although there are currently formidable regulatory barriers to this approach. In a research paper, G. Kasimatis et al. describe a comparative genomics study on the acne-derived type IA_1_ strain, HL096PA1, which they found harbours the first described linear plasmid in *P. acnes*, pIMPLE-HL096PA1. Homologues of pIMPLE-HL096PA1 appear to be widely distributed in ribotype 4 and 5 strains of *P. acnes* which are tetracycline resistant and associated with acne [11]. These plasmids carry a tight adhesion locus (Tad) that may enhance binding to host cell surfaces. Identification of this novel plasmid also provides a potentially new opportunity for genetic manipulation of *P. acnes* and possibly targeted therapy against specific disease-associated strains. HL096PA1 also contains fivefold more pseudogenes than the type IB strain KPA171202 to which it was compared, as well as island-like regions unique to the strain; type IB strains have rarely been isolated from acne lesions. Collectively, this paper provides additional data on *P. acnes* virulence properties and host adaptation mechanisms that may be relevant to our understanding of acne pathogenesis. Still on the theme of acne, E. A. Eady et al. in a second review paper hypothesise that the availability of water and possibly one or more water soluble micronutrients limits microbial growth within the follicular microenvironment of the skin and that reduction of water activity with biocompatible solutes of very high solubility may represent a novel and safe therapeutic approach in acne management. In an era of growing antibiotic resistance and the continuing widespread use of oral and topical antibiotics for the first line treatment of acne, the development of alternative therapeutic approaches for this extremely common skin condition is highly desirable.

In this special issue, we also have a research paper by N. Fischer et al. which describes a study into the intracellular fate of *P. acnes* in macrophages. This is an important study as the ability to persist in macrophages and other cell types as an intracellular pathogen may be important in the context of *P. acnes*-related diseases, particularly acne, sarcoidosis, and prostate cancer, to which the bacterium has been associated. This study confirmed the ability of *P. acnes* to survive phagocytosis *in vitro*, although no intracellular replication or escape from THP-1 host cells were observed. There was also no evidence of the *P. acnes*-containing phagosome fusing with lysosomes. The association of *P. acnes* with sarcoidosis, a systemic granulomatous disease, is extensively reviewed in a paper by Y. Eishi. While sarcoidosis is a disease of unknown etiology, infectious granulomas are commonly caused by intracellular pathogens and *P. acnes *is currently the only microorganism isolated from sarcoid lesions by culture. The review proposes mechanisms of granuloma formation in response to *P. acnes* in subjects with sarcoidosis and introduces a new concept of endogenous infection caused by hypersensitivity to indigenous bacteria. Interestingly, acne is also a delayed type hypersensitivity reaction, and if the condition is left untreated granuloma formation can occur [13]; this is rarely seen today due to clinical intervention. In a research paper by I. Dekio et al., the intracellular proteomes of different phylogroups were investigated and variations in protein expression profiles between strains grown under different oxygen tensions were examined. A good correlation was observed between genomic and proteomic profiles for the major genetic divisions of *P. acnes*. Differences were also noted in the intracellular proteome of strains grown under anaerobic/microaerophilic and aerobic culture conditions, which could be relevant in the context of *P. acnes* transferring from the skin surface to hypoxic/anoxic environments associated with deep seated systemic infections.

The role of *P. acnes* as a cause of infections related to indwelling medical devices is now increasingly recognised, especially in relation to prosthetic shoulder implants [2, 14]. A study from the Mayo Clinic, described by M. J. Karau et al., highlights how application of a vortexing-sonication technique for the detection of bacterial biofilm identifies *Propionibacterium *species, likely *P. acnes*, as common colonizers of breast tissue expanders. The presence of these colonised expanders in clinically uninfected subjects may present an increased risk factor for future infection or capsular contracture. The role of *P. acnes* in a wide range of implant-associated infections, along with its clinical management, is further discussed in a review by M. Portillo et al. They also describe how sonication of explanted prosthetic material improves the diagnosis of such infections and suggest that molecular methods may further increase the sensitivity of *P. acnes* detection in the resulting sonicate. In a paper by J. Rollason et al., *P. acnes* isolates from surgically excised lumbar disc herniations are characterised for phylogroup and antimicrobial susceptibility. Previous studies have suggested a role for *P. acnes* in the pathophysiology of disc degeneration and herniation, and antibiotic treatment does appear to have a positive effect upon patients with chronic back conditions [15–17]. In this study, type II and III strains were the predominant phylotypes isolated. Since strains from these genetic divisions are infrequently recovered from the skin, this study suggests that the recovery of *P. acnes* from excised disc material should not be readily dismissed as skin contamination in all cases. Furthermore, isolates from excised discs were susceptible to a range of antimicrobials commonly used to treat *P. acnes*-related infections.

In summary, this special issue covers a range of diverse topics related to *P. acnes* and disease, thus highlighting the widespread opportunistic behaviour of the bacterium in relation to infection. We hope the papers published will serve to further highlight the pathogenic potential of *P. acnes*, as well as stimulating further research into the role of the bacterium in human health and disease. In particular, future studies aimed at providing firm evidence of a role in chronic diseases, such as prostate cancer and disc disease, may provide exciting new opportunities for novel patient treatment and management strategies.
